# Machine Learning-Based Intelligent Scoring of College English Teaching in the Field of Natural Language Processing

**DOI:** 10.1155/2022/2754626

**Published:** 2022-08-04

**Authors:** Wei Wang

**Affiliations:** Changchun Guanghua University, Changchun 130031, Jilin, China

## Abstract

The current education evaluation is limited not only to the mode of simplification, indexing, and datafication, but also to the scientific nature of college teaching evaluation. This work firstly conducts a theoretical analysis of natural language processing technology, analyzes the related technologies of intelligent scoring, designs a systematic process for intelligent scoring of college English teaching, and finally conducts theoretical research on the Naive Bayesian algorithm in machine learning. In addition, the error of intelligent scoring of English teaching in colleges and universities and the accuracy of scoring and classification are analyzed and researched. The results show that the error between manual scoring and machine scoring is basically about 2 points and the minimum error of intelligent scoring in college English teaching under machine scoring can reach 0 points. There is a certain bias in manual scoring, and scoring on the machine can reduce the generation of this error. The Naive Bayes algorithm has the highest classification accuracy on the college intelligent scoring dataset, which is 76.43%. The weighted Naive Bayes algorithm has been improved in the classification accuracy of college English teaching intelligent scoring, with an average accuracy rate of 74.87%. To sum up, the weighted Naive Bayes algorithm has better performance in the classification accuracy of college English intelligent scoring. This work has a significant effect on the scoring of the college intelligent teaching scoring system under natural language processing and the classification of college teaching intelligence scoring under the Naive Bayes algorithm, which can improve the efficiency of college teaching scoring.

## 1. Introduction

As a key link of undergraduate teaching quality evaluation and the implementation of education evaluation reform, college students' evaluation of teaching quality is trend of high-quality development of higher education [1]. However, in recent years, there appear increasingly serious administration and formalization, which deviates from the original intention of mutual teaching and learning [2, 3]. Through policy analysis, teaching practice, and data simulation, it was found that some of the problems that have hampered the evaluation reform are expected to be solved by technological means [4, 5]. Student evaluation of teaching is an important part of the teaching quality guarantee system of colleges and universities. Its original intention is to promote the teaching development of teachers and improve the learning efficiency of students and then fundamentally guarantee the quality of classroom teaching and the effect of talent training. Meanwhile, effective student evaluation of teaching is also an important reference for the educational reform of colleges and universities [6, 7]. With the opening of the new round of undergraduate teaching evaluation in 2021, the reform of undergraduate teaching and evaluation has once again become an important focus for high-quality talent cultivation system in the 14th five-year plan period [8]. The Implementation Plan for the Examination and Evaluation of Undergraduate Education and Teaching in Ordinary Institutions of Higher Learning (2021–2025) proposes clearly promoting the reform of undergraduate education and teaching to avoid unscientific education evaluation and ensuring the priority of talent training and the core position of undergraduate education and teaching. By making good use of the organic integration of technology and educational governance, the quality of undergraduate education and teaching can be effectively improved so that students' evaluation of teaching can be credible, feasible, available, and popularized [9].

Some foreign researchers used Hadoop distributed architecture to explore and analyze students' behaviors by collecting data on social platforms [10]. Panessai et al. used data mining to explore the data of teaching evaluation process and established a teaching evaluation prediction model using Iterative Dichotomizer 3(ID3) decision tree algorithm. During this period, association rule algorithm is used to conduct comparative analysis of experimental verification results to explore the reliability of ID3 decision tree model [11]. Pliakos et al. estimated students' learning ability based on the learning information of students through machine learning algorithm and applied item response theory to predict response of the model. By combining the machine learning algorithm and item response theory, it was found that the estimation accuracy of students' learning ability was higher [12]. Kaempf and Finn predicted the feature terms of students' learning ability through diversified questions on the MOOC platform, aiming at solving the problem of slow improvement of students' learning ability [13]. Yuan studied the effectiveness of English composition scoring on the Kouku Correcting website and found that the evaluation results of the machine scoring system were consistent with those of the manual scoring system [14]. Herbart's theory divides interest into four stages: attention, expectation, method, and behavior. According to the psychological changes of students' activities, he puts forward the theory of teaching form stage, which includes teaching methods and teaching forms [15]. Domestic researchers applied machine learning to the teaching evaluation system, modelled the teaching evaluation model of ethnic colleges and universities through the relevant theories of artificial neural network, and conducted quantitative analysis of comprehensive indicators in a quantitative way. It was found that the neural network model based on BP (Back propagation) algorithm can obtain reasonable evaluation and analysis results for teaching evaluation [16, 17].

In this work, natural language processing (NLP) technology is firstly discussed to pave the way for intelligent scoring of college English teaching under machine learning. Then, related technologies of intelligent scoring are briefly introduced. Based on the principles and evaluation standards of intelligent evaluation of college English teaching, the system process of intelligent scoring of college English teaching is designed. Finally, the Naive Bayes (NB) algorithm in machine learning is theoretically studied. To verify the feasibility of this work, the error of intelligent scoring in college English teaching and the accuracy of scoring classification are analyzed and studied. The novelty of this work is that the Bayesian algorithm is applied to the intelligent scoring system of English teaching in colleges and universities, so that the evaluation of efficient English teaching can be truly intelligent. The objective of this work is to apply the machine learning technology to the field of English teaching, to provide a realistic basis for improving the overall teaching level of English teaching.

## 2. Relevant Technical Theory of Intelligent Grading of English Teaching in Colleges and Universities

### 2.1. Natural Language Processing

**Figure 1:**
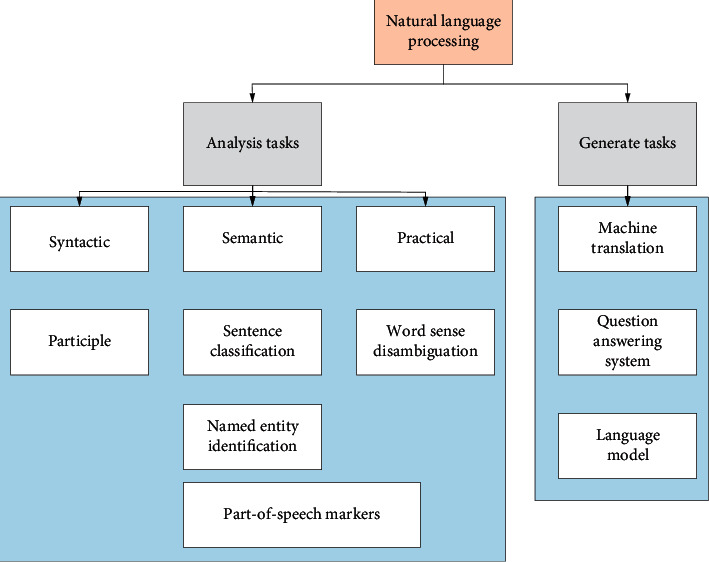
Task analysis and task generation under NLP.

NLP is to develop applications or services that can understand human language. It is practically applied to speech recognition, speech translation, understanding complete sentences, understanding synonyms of matching words, and generating grammatically correct complete sentences and paragraphs. The computer is used as a tool to process various technologies for written or oral forms.  Figure 1 shows the task analysis and task generation under NLP.

**Figure 2:**
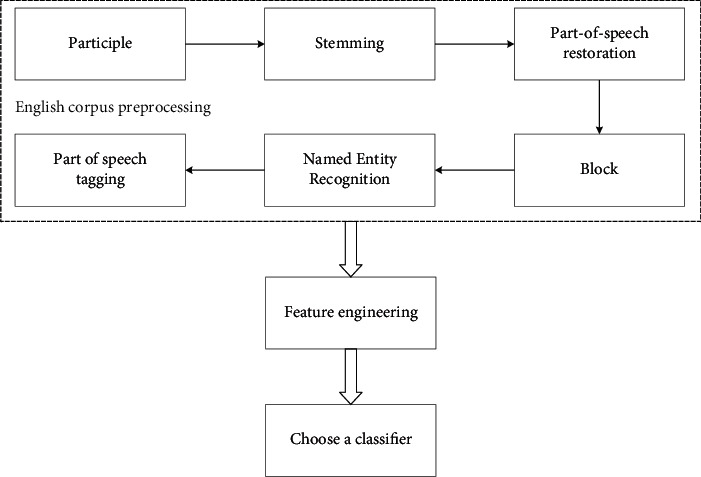
NLP process under machine learning.

In Figure 1, word segmentation is the division of a text into the smallest semantic units, i.e., words. For different languages, there are various basic vocabularies and grammars; word disambiguation: it is the process of selecting the correct meaning of a word from multiple meanings in the same context in natural language; named entity recognition: it is the process of extracting entities from a given text. The so-called entities are nouns such as person, place, and organization; lexical tagging: it is to classify a word into one of the different lexical categories such as noun, verb, adjective, and adverb; sentence classification: it is to understand whether a passage expresses a positive or negative meaning, such as teaching evaluation, to distinguish whether it is a good or bad review. It can be seen as a classification task; language generation: based on a text library, it can generate new text. For example, using Jin Yong's martial arts novels for training, the computer automatically generates Jin Yong style martial arts novels; question and answer system: typical application is Apple's Siri (Speech Interpretation and Recognition Interface) voice assistant. It can directly answer and solve the user's problems; machine translation (translation from one language to another): it requires the computer to understand its meaning first, before representing it in another language.  Figure 2 shows NLP process under machine learning.

As illustrated in Figure 2, for natural language processing under machine learning, the English corpus is first preprocessed, that is, word classification, and then part-of-speech recovery, named entity recognition and part-of-speech tagging. Secondly, according to feature extraction and feature selection, an appropriate classifier is selected to complete the language classification process. To process natural language with computer, the first thing to do is to represent text with appropriate data. In the early stage, NLP trains models by machine learning, and the text representation is similar to feature engineering in machine learning. Translating an English text into a “word bag” is a strategy to express the text according to the frequency of words. For example, first extract all the words in the text “Written language tends to use nouns instead of verbs” and “Written language tends to use nouns” to form a vocabulary, according to the vocabulary, (“Written,” “language,” “tends,” “to,” “use,” “nouns,” “instead,” “of,” “verbs”), and then use the frequency of each word to represent the corresponding text. The frequency of each word represents the corresponding text. In addition to the number of times a word appears, there can be other coding methods, such as whether a word appears or not, with 0 indicating no appearance and 1 indicating appearance. This method is called One-Hot, that is, one-hot coding [18]. You can also adopt the strategy of TF-IDF (term frequency, inverse document frequency) [19, 20], in which TF (Term frequency) is called word frequency, and its importance is shown in

(1) $$\begin{array}{c} tf_{i,j}=\frac{n_{i,j}}{\sum_{k}n_{k,j}}. \end{array}$$

In (1), *n*_*i*,*j*_ is the frequency of *d*_*j*_ that is the word appearing in the document *d*_*j*_, ∑_*k*_*n*_*k*,*j*_ is *d*_*j*_ that is the sum of the times of all words appearing in the document *d*_*j*_, and *tf*_*i*,*j*_ is the importance of words *t*_*i*_ in a specific document. IDF (inverse document frequency) is a method of weighting word frequency, expressed as

(2) $$\begin{array}{c} idf_{i}=\log\frac{\left|D\right|}{\left|\left\{j:t_{i}\in d_{j}\right\}\right|}, \end{array}$$

where |*D*| is the total number of |{*j* : *t*_*i*_ ∈ *d*_*j*_}| files in the corpus, |{*j* : *t*_*i*_ ∈ *d*_*j*_}| is the number of files containing words *t*_*i*_, and *idf*_*i*_ is the frequency of reverse files. If the word is not in the corpus, the dividend will be 0, so 1+|{*j* : *t*_*i*_ ∈ *d*_*j*_}| is generally used. Calculation of the high word frequency of a particular file and the low file frequency of the word in the whole file set can produce *tfidf*_*i*_ with a high weight, and the *tfidf*_*i*_ calculation expression is

(3) $$\begin{array}{c} tfidf_{i}=tf_{i}*idf_{i}. \end{array}$$

### 2.2. Related Technologies for Intelligent Scoring

**Figure 3:**
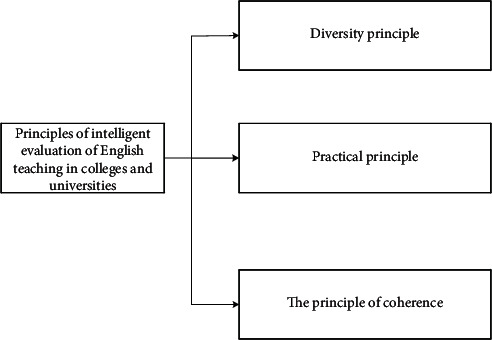
Intelligent evaluation principles of college English teaching.

More and more colleges and universities regard student evaluation as an important mechanism of teacher evaluation. This mechanism is aimed at arousing the enthusiasm of students to participate in classroom teaching, encouraging teachers to make full preparations and give lectures seriously, and urging teachers to pay attention to the teaching effect. The original intention of this mechanism is worthy of affirmation and has also received good results, but there are still some areas to be improved in the teaching practice, for example, one-size-fits-all, unification, and formalization. These deficiencies restrict the improvement of teaching quality and scientific research level to some extent. In terms of its negative impact on the field of economics, some colleges and universities pay too much attention to those scholars who teach modern mainstream economics and not enough attention to other types of scholars when introducing and assessing talents. Some “oratory” teachers are popular, while “thinking” scholars who are good at quiet thinking tend to get lower marks.  Figure 3 shows the intelligent evaluation principle of college English teaching.

**Figure 4:**
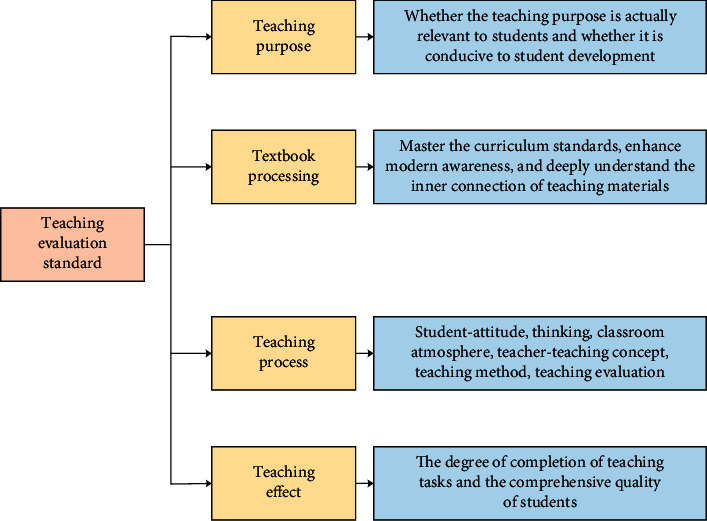
Evaluation criteria for college English teaching.

In Figure 3, the intelligent evaluation principle of English teaching in colleges and universities includes three parts, namely, the principle of diversity, the principle of practicality, and the principle of coherence. The plurality principle means that the intelligent English teaching not only should be limited to the evaluation of students, but also needs to introduce the participation of professional English teaching researchers to diversify teaching effect evaluation. The principle of practicability means that, according to the current evaluation system of English teaching in colleges and universities, there are many shortcomings. Practicability requires not only the improvement of students' and teachers' English ability, but also the comprehensive consideration of students' professional English ability and oral English level. Consistency principle suggests that evaluation of English teaching in colleges and universities should not have time limitations. It must follow the teachers' teaching theories and methods and make the students have a basic understanding of the English learning ability. At the same time, the teaching evaluation not only exists in classroom teaching, but also is performed through practice and activities.  Figure 4 shows the evaluation criteria of college English teaching.

**Figure 5:**
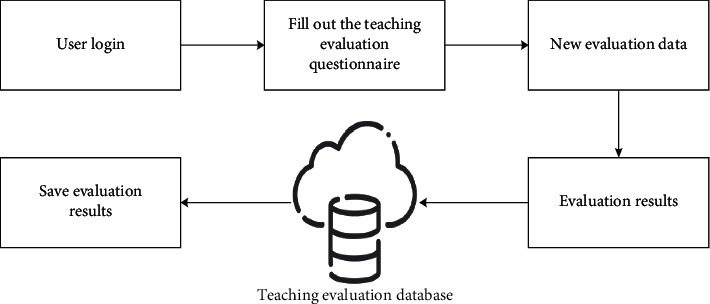
The systematic flow of intelligent grading of English teaching in colleges and universities.

College English teaching is evaluated in terms of teaching purpose, teaching material processing, teaching process, and teaching evaluation based on the principle of comprehensive consideration and scientific practice. The evaluation results are divided into excellent (above 85 points), good (84–75 points), qualified (74–60 points), and unqualified (below 60 points).  Figure 5 is the system process of intelligent scoring in college English teaching.

In Figure 5, the intelligent scoring module of college English teaching is analyzed from the three modules of data collection, evaluation process, and data storage.

### 2.3. NB Classification

**Figure 6:**
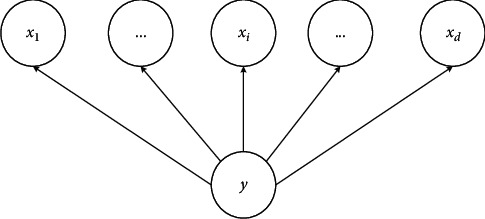
NB model.

Machine learning is essentially an approximation of a problem by a real model. Among them, supervised classification algorithm has been widely used in many business scenarios. Classification problem belongs to the prediction task, which is to obtain an objective function *f* through the learning of existing data sets and to map each attribute set *x* to the target attribute *y*. The *y* must be discrete. There are many methods to solve the classification problem. The basic classification methods mainly include decision tree, NB, artificial neural network, K-nearest neighbor, and support vector machine. In addition, there are ensemble learning algorithms for combining basic classifiers. Representative ensemble learning algorithms include random forest [21, 22]. In this work, the basic principles and advantages and disadvantages of each basic classifier are summarized.  Figure 6 shows the NB model.

The NB model in Figure 6 is a simple directed graph model that learns the probability of *y*(*x*) belonging to each classification [23–25]. *y* is the class variable, and *x*_1_, *x*_*i*_,…, and *x*_*d*_ are the attribute variables. First, the training sample set is defined in

(4) $$\begin{array}{c} \left(x_{1}^{\left(1\right)},x_{2}^{\left(1\right)},\cdots ,x_{n}^{\left(1\right)},y_{1}\right)\left(x_{1}^{\left(2\right)},x_{2}^{\left(2\right)},\ldots ,x_{n}^{\left(2\right)},y_{2}\right),\ldots ,\left(x_{1}^{\left(m\right)},x_{2}^{\left(m\right)},\ldots ,x_{n}^{\left(m\right)},y_{m}\right), \end{array}$$

where *m* represents the number of samples,  *x*_*i*_ is the feature vector of sample *X*, *n* is the sample feature, *y*_1_, *y*_2_, and *y*_*m*_ are the output variables, and the feature output has *k* categories, which are defined as *C*_1_, *C*_2_,…, *C*_*k*_. The sample number of each feature output category is *m*_1_, *m*_2_,…, *m*_*k*_. In the *k*th category, if the feature is discrete, the value of *x*_*i*_  category is *m*_*kjl*_, where *l* is 1,2,…, *S*_*j*_. *S*_*j*_ is the different values of feature *j*. The prior probability of *y* is calculated as

(5) $$\begin{array}{c} P\left(y=C_{k}\right)=\frac{m_{k}+\lambda }{m+k\lambda }, \end{array}$$

where *λ* is an arbitrary constant and the conditional probability distribution is expressed in

(6) $$\begin{array}{c} P\left(X=x|y=C_{k}\right)=P\left(X_{1}=x_{1},X_{2}=x_{2},\ldots ,X_{n}=x_{n}|y=C_{k}\right). \end{array}$$

The probability of determining the data set sample belonging to a certain category is expressed as

(7) $$\begin{array}{c} P\left(C_{j}|X\right)=\text{argmax}\frac{P\left(X|C_{j}\right)P\left(C_{j}\right)}{P\left(X\right)}. \end{array}$$

In (7), sample *X* of data set can be represented as {*x*_1_, *x*_2_,…, *x*_*n*_}, and *C*_*j*_ is the class label of the corresponding training data set. Assuming that the samples are independent from each other, the calculation expression of the category to which the data set belongs is shown in

(8) $$\begin{array}{c} P\left(X|C_{j}\right)=\prod_{i=1}^{n}P\left(x_{i}|C_{j}\right). \end{array}$$

From (8), *P*(*x*_1_*|C*_*j*_), *P*(*x*_2_*|C*_*j*_), and *P*(*x*_*n*_*|C*_*j*_) can be obtained, and then the category of test data set is deduced. Novendri et al. first adopted the idea of attribute feature weighting in NB classifier, improving the classification accuracy of NB method [26]. To take the correlation degree and accuracy coefficient of attributes into account at the same time, a new attribute calculation method is defined in

(9) $$\begin{array}{c} w_{i}=\frac{\left(w_{k}+w_{s}\right)}{2}, \end{array}$$

where *w*_*k*_ is the weighted coefficient of accuracy, *w*_*s*_ is the weighted coefficient of correlation degree, and *w*_*i*_ is the weight of total associated attribute. Then, NB assigned with a weight *w* is expressed as

(10) $$\begin{array}{c} P\left(X|C_{k}\right)=w*\prod_{i=1}^{n}P\left(x_{i}|C_{k}\right). \end{array}$$

**Figure 7:**
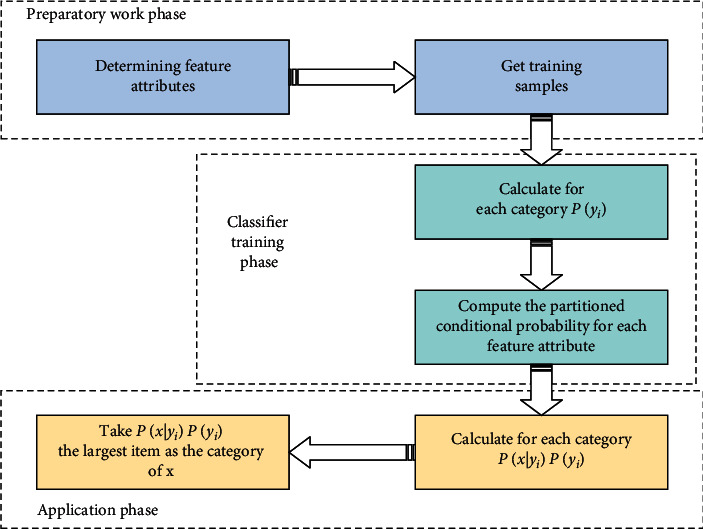
The classification process of NB.

The calculation process of NB classification is shown in Figure 7.

In Figure 7, the NB is divided into three stages. For the preparation stage, its task is to make necessary preparations for the NB classification. Its main work is to determine the attributes and characteristics of each attribute according to the specific circumstances. Then, each characteristic attribute is properly divided, and a part of the items to be classified are manually classified to form a training sample set. In this stage, all the data to be classified are input, and the output is characteristic attributes and training samples. The training stage of classifier is the generation of classifier. The main work is to calculate the occurrence frequency of each category in the training sample and the conditional probability estimation of each category based on the classification of each characteristic attribute and to record the results. Its input is characteristic attribute and training sample, and its output is classifier. This stage is mechanical and can be calculated automatically by the program according to the formula discussed earlier. In the application phase, its task is to classify items using classifiers. Its input is classifiers and items to be classified, and its output is the mapping relationship between items to be classified and categories. This stage is also mechanical and is done by a computer.

The data set used for the above model is the UCI data set IRIS. According to the needs of English teaching evaluation work, the above classification algorithm can be applied to the English teaching evaluation process. Taking a series of evaluation attribute values as input data and the comprehensive evaluation level as class label, learning a classifier through a certain classification algorithm can give the most likely class label for the new English teaching evaluation attribute value, that is, the evaluation result. In order to ensure the credibility of the evaluation results, it is necessary to select a suitable algorithm to construct the classifier. Accuracy is an indicator for evaluating the performance of a classifier, which is specifically defined as the ratio of the number of samples that the classifier can correctly classify to the total number of samples for a given test data set. The calculation equation is given as follows:

(11) $$\begin{array}{c} p=\frac{N^{\prime }}{N}. \end{array}$$

In (11), P represents the accuracy rate, N^′ represents the number of correctly classified samples, and N represents the total number of samples.

## 3. Results and Discussion

Based on the Nation Survey of Student Engagement, undergraduates in colleges and universities in Changchun were taken as the research subjects and graded according to the evaluation results of excellent, good, qualified, and unqualified. The evaluation content of college English teaching is the behaviors of teachers and students in the teaching process, as shown in Table 1.

### 3.1. An Analysis of Intelligent Scoring Errors in College English Teaching

**Figure 8:**
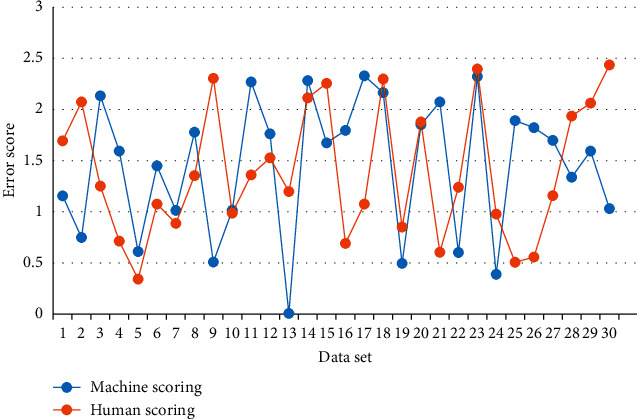
Intelligent scoring error in college English teaching.

Through NLP, the key words in the intelligent scoring process of college English teaching are extracted. For example, the scoring key words are good, not bad, understandable, abstract, and difficult. According to the score level, above 85 is excellent, between 84 and 75 is good, between 74 and 60 is qualified, and below 60 is not qualified. The error in the intelligent scoring process of Changchun colleges and universities is analyzed, and 30 score data sets are randomly selected on the Internet for testing.  Figure 8 shows the error of intelligent scoring in college English teaching.

Figure 8 shows the manual scoring and machine scoring under NLP of college English teaching. It is noted that the error curves of both manual scoring and machine scoring show irregular fluctuations, the error between manual scoring and machine scoring is basically less than 2 points, and the error of intelligent scoring of college English teaching under machine scoring can reach 0 points at the minimum. The mean test error is 1.41 points under manual scoring and 1.02 points under machine learning. According to the theory of human emotion, there is a certain deviation in the manual scoring, but the machine scoring can reduce this kind of error.

### 3.2. Classification Accuracy of Intelligent Scoring in College English Teaching Using Different Models

**Figure 9:**
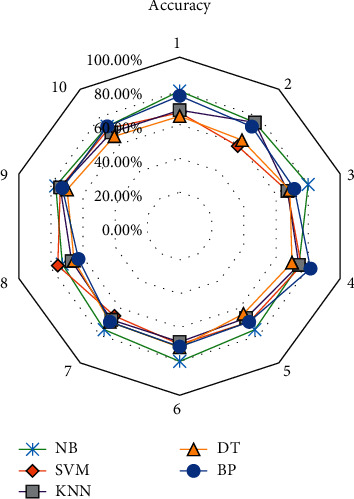
Classification accuracy of intelligent scoring in college English teaching using different models.

In this work, NB is compared with DT (decision tree), BP algorithm, KNN (K-nearest neighbor), and SVM. After 10 times of testing, the intelligent scoring data are classified.  Figure 9 shows the classification accuracy of intelligence scoring under different models.

**Figure 10:**
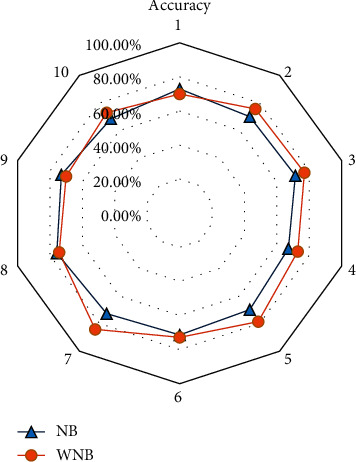
An analysis on the classification accuracy of college English teaching intelligence scoring based on WNB.

NB algorithm has the highest classification accuracy of 76.43% compared with SVM algorithm of 69.56%, KNN algorithm of 70.00%, DT algorithm of 67.37%, and BP algorithm of 72.27%. It can be concluded that it is reasonable to choose this algorithm in machine learning. By introducing weights into the NB algorithm, the classification accuracy before and after weighting is compared. The classification accuracy under weighted Naive Bayes (WNB) is shown in Figure 10.

It can be clearly seen from Figure 10 that the WNB algorithm improves the classification accuracy of intelligent scoring in college English teaching, with an average accuracy of 74.87%, while the NB algorithm has an average classification accuracy of 71.29%. Above, the WNB algorithm has a good performance in the classification accuracy of intelligent scoring of college English teaching.

Lin applied the classification algorithm in machine learning to the construction of the evaluation model, which further improved the scientificity and feasibility of teaching evaluation. In addition, the empirical algorithm was used as the basic algorithm to evaluate the teaching quality, and the subject word distribution obtained by the joint model training was used as the original knowledge. The scoring of the research model was close to the standard manual scoring, which can provide a theoretical reference for subsequent related research [27]. This is similar to the research in this work, which can provide a basis for college teaching. Sun et al. established an English teaching evaluation implementation model based on machine learning decision tree technology. It provided valuable data from a wide range of information, summarizing rules and data to help teachers improve education and students' English achievement. This system embodied the idea of artificial intelligence expert system. Test applications showed that the system can help students improve their learning efficiency and make learning content more relevant [28]. This is similar to the results of this work, which can also apply machine learning to English teaching. This shows that the integration of cutting-edge technologies (such as machine learning technology under artificial intelligence) in college teaching, especially English teaching, is crucial to the development of English education.

## 4. Conclusions

In the work, the NB algorithm is used to study the classification accuracy of intelligent scoring in college English teaching, and the error of intelligent scoring in C=college English teaching under NLP is analyzed. According to the analysis of the research results, the average test error under manual scoring is 1.41 points, while the average test error under machine learning is 1.02 points. According to the theory of human emotion, there is a certain deviation in the manual scoring, but the machine scoring can reduce this kind of error. The average classification accuracy of NB algorithm is 76.43%, higher than SVM algorithm of 69.56%, KNN algorithm of 70.00%, DT algorithm of 67.37%, and BP algorithm of 72.27%. The WNB algorithm has an average accuracy of 74.87% in intelligent scoring classification of college English teaching, and that of NB algorithm is 71.29%. In conclusion, the WNB algorithm has a good performance in the classification accuracy of intelligent scoring. This study plays a significant role in the scoring of intelligent teaching scoring system in colleges and universities under NLP and the classification of intelligent teaching scoring, which can improve the work efficiency of teaching scoring in colleges and universities and evaluate the teaching process scientifically to a certain extent. However, the number of data sets selected is limited, and the researchers could increase the amount of data, so as to strengthen the findings of the study.

## Figures and Tables

**Table 1:** The content of college English teaching evaluation.

The subjects	Content
Teacher	Classroom atmosphere, homework completion, teaching content extension, etc.
Student	Learning initiative, awareness of cooperation and communication, learning attitude, etc.

## Data Availability

The data that support the findings of this study are available from the corresponding author upon reasonable request.
